# Dr. Mary Walker

**Published:** 1890-06

**Authors:** 


### DR. MARY WALKER.

"How did I happen to take up the dress reform costume?" said Dr. Mary Walker, the other day; "I will tell you. When I was a little girl I became interested in a lot of medical books which my father had in the house. That was at Oswego, N. Y., fifty years ago. My father had once studied medicine, though he never practiced. He was a dress reformer. He believed in hygienic food, in sanitary dress, in many things which the people of that day knew little about. From him and from my familiarity with the laws of health drawn from the medical books, I received the inspiration to take up the dress reform. I never wore a corset, nor did any of my four sisters, while we were at home. My parents supported me in my determination to show the world that I was in earnest, that I had the courage of my convictions, by wearing the reform suits. I will never forget the day I first appeared on the street in one of my own costumes. It was not as mannish as some suits I have since worn, but it was in fear and trembling that I opened the door of our house and started for the sidewalk. I was faint and dizzy, and nothing but the sheer exercise of will power kept me on my feet or continued my limbs in motion.

The next day it was a little easier, but though the world has thought me hardened and brazen, I have never seen the day when it was not a trial to me to appear in public in a reform dress. Every jeer has cut me to the quick. Many times have I gone to my room and wept after being publicly derided. No one knows, or will ever know, what it has cost me to live up to my principles, to be consistent with my convictions and declarations; but I have done it, and am not sorry for it. For nearly thirty-five years I have worn some form or other of reform suits. I have experimented with different garments. and have worn every thing from blouses and bifurcated trousers to a man's regular outfit, high hat and all." And here the doctor, who is now lying seriously ill at Washington, smiled rather grimly at the tall hat hanging on the bureau, conscious that she may never again have use for it.—*Woman's News.*

